# The genetics of cannabis lifetime use

**DOI:** 10.1038/s41386-025-02255-4

**Published:** 2025-10-03

**Authors:** Uri Bright, Sarah Beck, Marco Galimberti, Priya Gupta, Yu Chen, Cecilia Dao, Yaira Z. Nunez, Henry R. Kranzler, Yu Zhou, Yingzhe Zhang, Karmel W. Choi, Daniel F. Levey, Joel Gelernter

**Affiliations:** 1 Department of Psychiatry, Yale School of Medicine, New Haven, CT USA; 2 Veterans Affairs Connecticut Healthcare System, West Haven, CT USA; 3 Department of Psychiatry, University of Pennsylvania Perelman School of Medicine, Philadelphia, PA USA; 4 Crescenz Veterans Affairs Medical Center, Philadelphia, PA USA; 5 Department of Psychiatry, Massachusetts General Hospital, Boston, MA USA; 6 Department of Epidemiology, Harvard T.H. Chan School of Public Health, Boston, MA USA; 7 Departments of Genetics and Neuroscience, Yale School of Medicine, New Haven, CT USA

**Keywords:** Human behaviour, Genetics

## Abstract

Cannabis is one of the most commonly used drugs in the world, and use is trending alarmingly higher. We aimed to examine the genetic basis of cannabis lifetime use (CanLU) and its genetic relationships with a variety of psychiatric- and physical health-related phenotypes. We conducted a multi-ancestral genome-wide association study (GWAS) of CanLU using data from All of Us in five genetic populations. We meta-analyzed the results of EUR participants with previously published CanLU data (total effective sample size: 258,823), and conducted a set of post-GWAS analyses, including genetic correlation analysis using LDSC, local genetic correlation analysis with LAVA, Mendelian randomization (MR) to assess causality, and phenomewide association analysis. We found 11 independent variants significantly associated with CanLU, most prominently CADM2*rs7609594 (*p* = 7.4 × 10^−20^). CanLU was genetically correlated with traits related to openness to experience and risk taking, including substance use and sexual behaviors. MR demonstrated that most of these traits had a bidirectional causal relationship with CanLU, and six were locally genetically correlated with CanLU in a region that maps to *CADM2*. Genetic correlations sometimes differed from those previously observed for cannabis use disorder. Our results highlight the distinct genetic architecture of CanLU, and support the genetic, and biological, differentiation between CanLU and cannabis use disorder. Genetic correlations between CanLU and other risk taking- and substance use-related traits indicate a broad mutual genetic mechanism underlying these traits, and suggest involvement of *CADM2*. These findings provide potential targets for future prevention and intervention strategies for substance use and risk-taking behaviors.

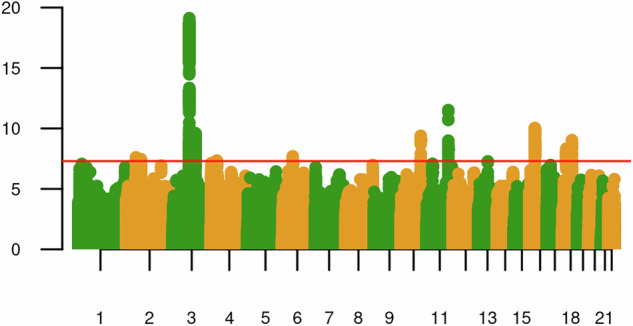

## Introduction

Cannabis is one of the most commonly used drugs worldwide. In the United States (US), among daily or near-daily consumers, consumption rates of cannabis exceed even those of alcohol (17.7 million vs 14.7 million Americans in 2022) [1], and >10% of the adult population reported having used cannabis in the last 30 days [2, 3]. A 2022 report showed that 27.3% of Europeans >age 15 reported cannabis lifetime use (CanLU) [4]; in 2023, 29.3% of adults age 35–50 reported cannabis use in the past year [5]. These numbers have been increasing since the mid-1990s [1] due to medicalization, decriminalization, legalization and commercialization processes that have occurred around the world, especially in Europe [6] and North and South America [7–9]. Nevertheless, recreational use of cannabis is still illegal in most of the world, including the US (on a federal level). While cannabis use has been relatively common for thousands of years [10], research regarding its biology and its effects on humans became abundant relatively recently. Various studies point to the effects of cannabis use on brain development during adolescence [11] and to long-term effects of cannabis on brain structures and on measures of brain function [12, 13]. Much work remains regarding the effects of cannabis and the genetics underlying cannabis use, its consequences – which include cannabis use disorder (CanUD) – and the genetic relationships between cannabis use and other traits and diseases.

Three previous large-scale genome-wide association studies (GWAS) addressed the genetics of CanLU [14–16]. Despite differences, all showed significant association between CanLU and single nucleotide polymorphisms (SNPs) located at the *CADM2* gene, suggesting a role for its encoded protein – cell adhesion molecule 2 – in the biology of CanLU. *CADM2* was also associated with CanUD [17], and other substance use and risk taking traits [18–21]. Moreover, CanLU was found to be locally genetically correlated with substance use traits in a region on chromosome 3 that includes *CADM2* [22]. CanLU differs genetically from CanUD, with differing sets of genetic correlations with other traits [17]. Although many cannabis lifetime users are also lifetime smokers, there are distinct genetic differences between CanLU and different tobacco smoking traits, including smoking initiation [23].

We used GWAS and post-GWAS analyses to study the genetic basis of CanLU, and to investigate its relationships with other traits of interest. We include larger sample size than previous publications, and with access to multi-ancestral data. Specifically, we conducted GWAS of CanLU in populations of European (EUR), African (AFR), admixed American (AMR), East Asian (EAS) and South Asian (SAS) ancestries from the All of Us Research Program (AoU) [24]. We then meta-analyzed the EUR results with summary statistics of CanLU in subjects from the International Cannabis Consortium (ICC) and the UK Biobank (UKBB) (where only EUR data are available) [14], as well as findings from all five ancestries meta-analyzed. We calculated the genetic correlations between CanLU and selected traits of interest using LDSC and phenome-wide association study (pheWAS) analyses, and assessed the causation between these traits (using Mendelian Randomization; MR), transcriptome-wide association study (TWAS) and fine-mapping; we also calculated the genetic correlations with various brain measures and the local genetic correlations (using LAVA) between CanLU and traits of interest.

## Materials and methods

### Cohorts

This study used anonymized publicly available data and it is not considered human subjects research. Our primary analysis used whole-genome sequencing data from AoU, version 7. Genotyping and quality control procedures were described previously [25]. CanLU was defined using a lifestyle survey; participants whose response for the question “In your lifetime, which of the following substances have you ever used?” included “Marijuana” were considered as cases. Participants who completed the lifetime survey and whose answer for the same question did not include “Marijuana” were assigned as controls. 188,438 participants were included (103,130 cases), divided according to five ancestries based on genomic data: EUR, AFR, AMR, EAS, SAS [25]. PCs were calculated for each ancestry separately using PLINK 2.0. Participants’ age was defined by the year of consent (i.e., to join the AoU program) minus the year of birth. We also included a sex-stratified analysis for EUR (but not other ancestries due to low power; Supplementary Table S1). After analysis, we conducted an EUR meta-analysis (63,300 cases) of AoU results with summary statistics of a previous study [14], which include 35,297 subjects from ICC (15,107 cases) and 126,785 subjects from UKBB (28,273 cases).

### Genome-wide association study (GWAS) analysis and meta-analysis

After kinship removal with kinship coefficient cutoff of 0.1 (raw sample size, before kinship removal, is presented in Supplementary Table S1), based on previously published relatedness data [25], GWAS was conducted for all five AoU ancestries using logistic regression in PLINK 2.0, with the first ten genetic PCs, sex, and age as covariates. Variants with minor allele frequency (MAF) < 0.1% and Hardy-Weinberg equilibrium (HWE) *p* < 1 × 10^−6^ were excluded. All-EUR and cross-ancestry meta-analyses, weighted by effective sample size, were performed using METAL [26]. Heterogeneity across samples was calculated. In all analyses we applied a standard genome-wide multiple testing correction (*p* < 5 × 10^−8^). The association results were visualized using the R package qqman [27].

### MAGMA gene-based analyses

We used MAGMA [28], implemented in the FUMA platform [29] to conduct a gene-based analysis of the EUR and cross-ancestry meta-analyses. Input SNPs were mapped to 19,019 protein-coding genes in EUR and 18,994 cross-ancestry (statistical significance threshold: *p* = 2.63 × 10^−6^).

### LDSC and SNP-based heritability

We performed linkage disequilibrium score (LDSC) regression based on the linkage disequilibrium (LD) reference from the 1000 Genomes data [30] for all EUR cohorts, and calculated SNP heritability for CanLU. We investigated the genetic correlation between CanLU and 43 psychiatric disorders, substance use traits, behavioral phenotypes, personality traits and general health characteristics [17, 19, 23, 31–45]. Because many cannabis lifetime users are also lifetime smokers, we compared lifetime use of cannabis with that of lifetime tobacco smoking. Specifically, in the AoU sample included here, 54% of the EUR CanLU subjects also reported smoking more than 100 cigarettes lifetime. We calculated the genetic correlation between lifetime smoking and CanUD with all traits included in the analysis, and tested the difference between all pairs using a Wald test, accounting for the covariance between the estimates (with Bonferroni correction for 86 comparisons). After Bonferroni correction for 129 genetic correlation tests (0.05/129), the statistical significance threshold was set at *p* = 3.88 × 10^−4^. Genetic correlations were also calculated between the EUR meta-analysis of CanLU and 2126 measures of brain structure and function [46] (significance threshold after Bonferroni correction: *p* = 2.35 × 10^−5^).

### Cross-ancestry genetic correlations

We used Popcorn [47] to calculate the cross-ancestry genetic correlations for CanLU in EUR vs AFR and AMR populations. Due to a limited sample size, we did not include participants of EAS and SAS ancestries in this analysis.

### Local genetic correlations

We used LAVA [48] to calculate local genetic correlations between the EUR meta-analysis of CanLU and 43 psychiatric disorders, substance use traits, behavioral phenotypes, personality traits and general health characteristics in EUR, hypothesized to be related to CanLU. The genome was divided into 2495 genomic regions, which provides minimum LD between the region and maintains an approximately equal size of the regions of ~1 MB. Breakpoints between regions were computed according to the LD between neighboring SNPs as described in ref. [48], to maintain regions as relatively independent. Univariate local correlations were calculated for each trait. Bonferroni correction for 2495 regions (i.e. 2495 tests) yielded a statistical significance threshold of *p* = 2 × 10^−5^ for local genetic heritability. Regions that reached significance were used to calculate genetic correlations with CanLU (18,103 regions in 43 traits). Local genetic correlations were calculated for all pairs of CanLU with other traits. Significance threshold after Bonferroni correction for 18,203 tests: *p* = 2.8 × 10^−6^.

### Mendelian randomization (MR)

All traits with significant genetic correlation (LDSC) and/or local genetic correlation (LAVA) with the CanLU EUR meta-analysis (a total of 22 traits), were selected for follow-up MR analysis. We used a *p*-value threshold of 1.0 × 10^−5^ to define genetic instruments. For all trait pairs, bidirectional tests were performed, i.e. CanLU was used both as exposure and outcome. MR was conducted using the MRlap package [49] in R 4.2.0 to account for possible overlap of the datasets. Clumping was conducted with LD threshold r^2^ = 0.05 and a window of 500 kb. To account for potential pleiotropy and violations of instrumental variable assumptions, we applied several complementary two-sample MR methods [50], including MR-Egger, weighted median, inverse-variance weighted (IVW), simple mode, and weighted mode. Significance threshold after Bonferroni correction for 264 tests (six methods, 22 traits, applying CanLU both as exposure and outcome): *p* = 1.89 × 10^−4^. Pleiotropy was tested for all significant causal effects using the MR-Egger intercept test.

### Transcriptome-wide association study (TWAS)

We conducted TWAS using GTEx_v8 [51], with expression data of 13 brain regions in samples of EUR ancestry. We used the 1000 Genomes dataset as an LD reference. Using FUSION [52], we identified associated genes, then processed the results to distinguish conditionally independent genes, using FUSION’s built-in post-processing script. In 13 tissues, there were a total of 57,350 genes measured - an average of 4411 (±1467) genes per tissue, with partial overlap (i.e., the same gene could be assessed in several tissues). After Bonferroni correction for 57,350 tests, *p*-value threshold was set 8.72 × 10^−7^. For each gene, we kept the tissue with the lowest TWAS *p*-value.

### Fine-mapping of genomic risk regions

We conducted fine-mapping of genomic risk regions using GTEx_v7 [51], with expression data of 13 brain regions in samples of EUR ancestry. We used the 1000 Genomes dataset as an LD reference. Using FOCUS [53] we identified possible causal genes for CanLU as those with a posterior inclusion probability (PIP) > 0.7.

### Phenomewide association study (PheWAS)

MGB Biobank (MGBB) samples were genotyped using Illumina’s Global Screening Array (GSA). We performed genotype data QC and computed genetic PCs (https://github.com/getian107/MGBB-QC). Following population assignment, we focused on EUR participants in the MGBB due to limited sample size for other ancestral groups in both the MGBB cohort and the discovery GWAS. In MGBB, 33,616 (71%) out of 47,321 participants were classified as EUR. EUR participants were excluded if there were inconsistencies between their reported and genetic sex or if they were outliers in absolute heterozygosity. Genotype imputation was performed using the Michigan Imputation Server with the Haplotype Reference Consortium reference panel. Post-imputation, we excluded markers with imputation quality INFO score <0.80, MAF < 0.01, significant deviation from HWE (*P *< 1 × 10⁻¹⁰), or missing call rate >0.02. The Phecode matrix included 1711 phecodes and the final target dataset included 30,701 unrelated EUR individuals. Significance threshold after Bonferroni correction for 1711 tests: *p* = 2.92 × 10^−5^. Additional details can be found in the GitHub (https://github.com/getian107/MGBB-QC). PRS for CanLU were computed using PRS-CS [54] and PLINK1.9 and standardized before conducting PheWAS. ICD-9 and ICD-10 diagnosis codes were extracted from the electronic health records and converted into phecodes. Individuals with at least two occurrences of a phecode were classified as cases, those with none were designated as controls, and those with one were excluded, due to possible unreliability of a single diagnosis. A total of 1711 phecodes with at least 20 cases were evaluated in the MGBB. PheWAS was performed using logistic regression for each phecode, utilizing the PheWAS package in R. The models included sex, age, and the top ten genetic PCs as covariates.

## Results

### GWAS

**Fig. 1:**
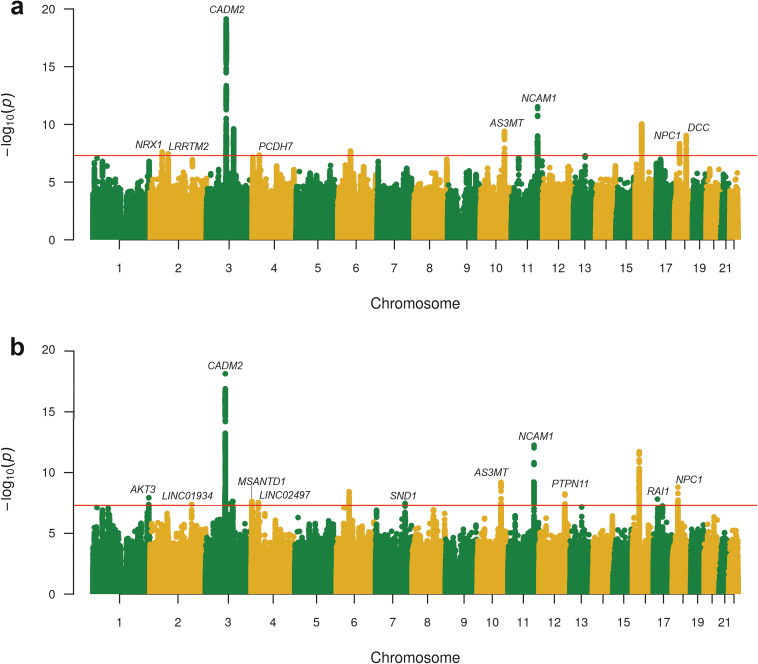
GWAS results. **a** AoU, ICC and UKBB European population GWAS meta-analysis of CanLU; **b** Cross-ancestry meta-analysis of CanLU (including EUR meta-analysis, AFR, and AMR, EAS and SAS subjects from AoU). Significant SNPs located within or nearby coding genes are annotated.

188,438 subjects from AoU were included in this study. In EUR, we identified one genome-wide significant (GWS) locus, CADM2*rs1821351. The sex-stratified GWAS yielded no significant findings in either sex, nor were there GWS loci in the other ancestries (the top-500 SNPs in each analysis are presented in Supplementary Table S2; Manhattan and quantile-quantile (QQ) plots of all the individual ancestry GWAS in AoU are presented in Supplementary Figs. S1–S5). Meta-analysis in EUR combined data from the AoU analysis with summary statistics from a previous study [14], which included 35,297 participants from ICC (15,107 cases) and 126,785 participants from UKBB (28,273 cases) (23andme data EUR subjects were also included in this study; these data are not publicly available and were not included in our meta-analyses). In total, we assembled a sample of 271,134 subjects (106,680 cases) that were included in the GWAS analyses (Table 1). The lead SNP and 1080 other SNPs comprising of a total of 1247 variants that were statistically significant (*p* < 5 × 10^−8^), showed no heterogeneity (I^2^ = 0%, HetPVal >0.05). We identified 11 independent GWS SNPs for CanLU, including NRXN1*rs858931, LRRTM2*rs1446710, CADM2*rs7609594, rs861001, PCDH7*rs16867990, rs4712084, AS3MT*rs10883796, NCAM1*rs9919557, rs2106480, NPC1*rs1788783 and DCC*rs2045154 (Table 2, Fig. 1a). A cross-ancestry meta-analysis, which included the EUR meta-analysis and AFR, AMR, EAS, and SAS subjects from AoU, yielded a total of 15 independent GWAS SNPs. These included AKT3*rs10754807), LINC01934*rs11902472, CADM2*rs1821351, rs9858134, rs1604060, MSANTD1*rs3095075, LINC02497*rs112738674, rs9357822, SND1*rs2402870, AS3MT* rs10883796, NCAM1*rs10891480, PTPN11*rs74644204, rs62034323, RAI1*rs4925114 and NPC1*rs1623003 (Table 2, Fig. 1b). QQ plots of the EUR and cross-ancestry meta-analyses are presented in Supplementary Figs. S6, S7.

**Table 1:** Demographics.

Ancestry	Cohort	*n* Controls	*n* Cases	*n* Total	*n* Effective^a^
EUR	All of us	45,752	63,300	109,053	106,228
	ICC	20,190	15,107	35,297	34,565
	UKBB	98,512	28,273	126,785	87,873
	EUR meta analysis	164,454	106,680	271,135	258,823
AFR	All of us	18,000	24,599	42,600	41,577
AMR	All of us	18,597	13,025	31,623	30,640
EAS	All of us	2591	1615	4206	3980
SAS	All of us	1336	591	1928	1639
All	Cross-ancestry meta-analysis	204,978	146,510	351,492	341,762

*EUR* European, *AFR* African, *AMR* South American, *EAS* East Asian, *SAS* South Asian.

^a^Effective number was calculated by this formula: 4/(1/*n* Cases) + (1/*n* Contorls).

**Table 2:** Lead SNPs.

	rsID	CHR	Position (GRCh38)	*P*-value	Gene	Direction
AoU - EUR	rs1821351	3	85439175	2.15E−08	*CADM2*	−
EUR meta analysis	rs858931	2	50702091	2.44E−08	*NRXN1*	−
	rs1446710	2	76975621	3.53E−08	*LRRTM2*	+
	rs7609594	3	85433445	7.40E−20	*CADM2*	−
	rs861001	3	118001508	2.47E−10	−	−
	rs16867990	4	31128176	4.49E−08	*PCDH7*	+
	rs4712084	6	54996486	2.00E−08	−	−
	rs10883796	10	102895558	4.01E−10	*AS3MT*	+
	rs9919557	11	113006686	2.98E−12	*NCAM1*	−
	rs2106480	16	28526650	9.39E−11	−	+
	rs1788783	18	23581170	4.72E−09	*NPC1*	+
	rs2045154	18	52513306	9.21E−10	*DCC*	−
Multi-ancestry meta analysis^a^	rs10754807	1	243640856	1.2E−08	*AKT3*	−
	rs11902472	2	181126132	4.1E−08	*LINC01934*	+
	rs1821351	3	85439175	7.34E−19	*CADM2*	−
	rs9858134	3	101542135	3.8E−08	*−*	−
	rs1604060	3	118053189	2.4E−08	*−*	−
	rs3095075	4	3251456	2.4E−08	*MSANTD1*	−
	rs112738674	4	31184338	3E−08	*LINC02497*	+
	rs9357822	6	54962341	3.8E−09	*−*	−
	rs2402870	7	127798409	3.4E−08	*SND1*	−
	rs10883796	10	102895558	6.3E−10	*AS3MT*	+
	rs10891480	11	112959803	5.6E−13	*NCAM1*	−
	rs74644204	12	112425187	5.8E−09	*PTPN11*	+
	rs62034323	16	28525152	1.9E−12	*−*	−
	rs4925114	17	17807956	1.5E−08	*RAI1*	−
	rs1623003	18	23585199	1.6E−09	*NPC1*	+

^a^Includes the EUR meta-analysis and AFR, AMR, EAS and SAS subjects from AoU.

### MAGMA gene-based analyses

Using MAGMA gene-based analysis we found 23 significant genes associated with CanLU in EUR, four of which were GWS in the GWAS. (Supplementary Table S3). Cross-ancestry, we found 24 significant genes associated with CanLU (Supplementary Table S4). Seventeen of the genes had a significant effect in both analyses. The strongest effect in both was of *CADM2* (*p* = 1.78 × 10^−16^ in EUR, *p* = 3.78 × 10^−13^ cross-ancestry).

### Genetic correlations

**Fig. 2:**
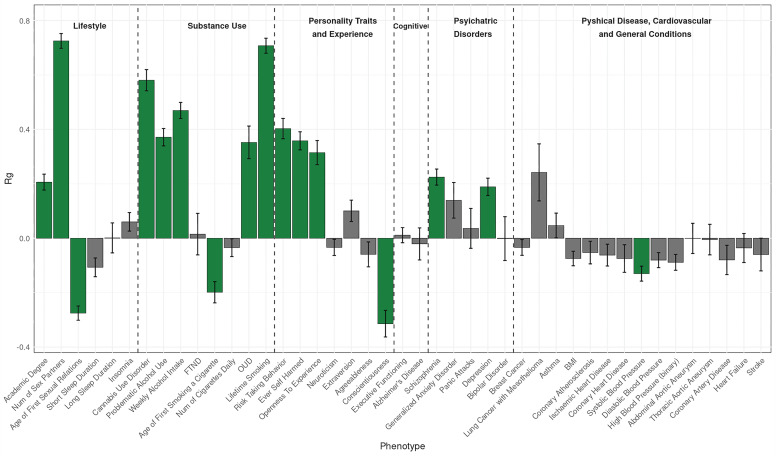
Genetic correlations between cannabis lifetime use and 43 traits of interest. Statistically significant effects are highlighted in green. Exact figures are presented in Supplementary Table S2.

The SNP-based heritability (h^2^) of CanLU in the EUR meta-analysis was 3.68% (h^2^ = 0.0368 ± 0.003) (Supplementary Table S5). LDSC was used to calculate the genetic correlation between the CanLU phenotypes as defined in AoU and the ICC/UKB meta-analysis [14], with r_g_ = 0.903 (±0.053). For genetic correlations with other traits, 16 traits (out of 43 tested) were significantly genetically correlated with CanLU (statistical significance threshold: *p* = 0.0004). CanLU was most highly positively correlated with number of sexual partners (r_g_ = 0.725 ± 0.027) (and negatively correlated with age of first sexual relations (r_g_ = −0.275 ± 0.026)), lifetime smoking (r_g_ = 0.707 ± 0.028), CanUD (r_g_ = 0.581 ± 0.039), weekly alcohol consumption (r_g_ = 0.469 ± 0.03), risk taking behavior (r_g_ = 0.403 ± 0.037), problematic alcohol use (PAU) (r_g_ = 0.371 ± 0.032), self-harm (r_g_ = 0.358 ± 0.033) and opioid use disorder (OUD) (r_g_ = 0.352 ± 0.06). CanLU was also genetically correlated with two personality traits: positively with openness to experience (r_g_ = 0.315 ± 0.044) and negatively with conscientiousness (r_g_ = −0.314 ± 0.049) (Fig. 2, Supplementary Table S6). We also calculated the genetic correlation between CanLU in males and females in AoU, with r_g_ = 0.915 ± 0.173, indicating that the genetic mechanism of CanLU is similar for the different sexes.

We next examined the genetic correlations between lifetime smoking, CanUD, and all the traits that were included in the genetic correlation analysis with CanLU. For most of the traits that had a significant r_g_ value with CanLU, there were also significant correlations with lifetime smoking and CanUD in the same direction, with a few exceptions: unlike CanLU, lifetime smoking was not significantly genetically correlated with openness to experience; having an academic degree was significantly genetically correlated with both traits, but the correlation with lifetime smoking was negative while with CanLU it was positive. Besides academic degree, four traits that were genetically correlated with both CanLU and lifetime smoking had significantly different r_g_ values with those phenotypes (lifetime number of sex partners, age of first sexual relations, age of first smoking a cigarette and depression). Several traits were correlated with lifetime smoking but not with CanLU; those included sleep-related traits, neuroticism, extraversion, lung cancer, BMI and several cardiovascular traits. Likewise, traits that were genetically correlated with CanUD but not with CanLU included lung cancer, asthma and cardiovascular traits. Similarly to lifetime smoking, the genetic correlation of CanUD with academic degree was negative (Supplementary Figs. S8, 9, Supplementary Table S6). Some of these differences likely reflect power differences between analyses.

We also assessed genetic correlations between CanLU and 2126 brain structure and function measures, with no significant findings (Supplementary Table S7).

### Cross-Ancestry Genetic Correlations

The genetic correlation between CanLU in EUR and AFR was 1.21 (±0.48; *p* = 0.03) and between CanLU in EUR and AMR was 0.85 (±0.21; *p* = 4.4 × 10^−5^).

### Local genetic correlations

Seventeen of the 43 traits studied (40%) had a statistically significant local genetic correlation with CanLU in at least one region (significant at a threshold of *p* = 2.8 × 10^−6^; Supplementary Table S8). Six traits were significant for the overall genetic correlation only, and eight traits were not significant for the overall genetic correlation but showed regional correlations with CanLU. Most prominently, 13 genomic regions were associated with Alzheimer’s disease (AD), nine negatively correlated and four positively correlated, which may help to explain the absence of an overall correlation using LDSC. Nine traits were significantly correlated with CanLU both globally (i.e. LDSC) and locally (i.e. LAVA).

For six of the 17 traits that were genetically correlated with CanLU, there was a significant genetic correlation with the region chr3:84,698,481:85,807,679. These traits include age of first sexual relations, number of sexual partners, CanUD, risk taking behavior, lifetime smoking, and weekly alcohol intake. *CADM2* is the only protein-coding gene that maps to this interval.

### Mendelian randomization

All traits with significant (at a threshold of *p* = 1.89 × 10^−4^) overall or local genetic correlations with CanLU (22 in total) were analyzed for MR using MRlap [49], with a *p*-value threshold of 1.0 × 10^−5^ to define genetic instruments (Supplementary Table S9). MR analyses inferred significant causal effects of CanLU (as exposure) on 11 of the analyzed traits, and a significant causal effect of 13 traits on CanLU (as outcome). Nine traits showed a bidirectional relationship, i.e., the two traits were causal for one another. The strongest causal effects of CanLU were on lifetime number of sex partners (effect_corrected_ = 0.553 ± 0.039, *p*_corrected_ = 4.35 × 10^−46^) and lifetime smoking (effect_corrected_ = 0.463 ± 0.039, *p*_corrected_ = 9.35 × 10^−32^). The strongest causal effects on CanLU were for CanUD (effect_corrected_ = 0.502 ± 0.059, *p*_corrected_ = 1.43 × 10^−17^), lifetime smoking (effect_corrected_ = 0.434 ± 0.024, *p*_corrected_ = 8.39 × 10^−72^) and lifetime number of sexual partners (effect_corrected_ = 0.433 ± 0.022, *p*_corrected_ = 2.32 × 10^−83^). While MRlap was used to account for sample overlap, additional methods were implemented to assess the robustness of the causal estimates under different assumptions, using Two-Sample MR [50]. All significant values from MRlap were in the same direction (using different MR methods) (Supplementary Table S10). We also tested horizontal pleiotropy between traits, and found potential horizontal pleiotropy, with low MR-Egger intercept values but statistically significant, between CanLU and self-harm, lifetime smoking, lifetime number of sex partners, PAU and weekly alcohol intake (Supplementary Table S11). Therefore, MR results regarding the causality between these traits and CanLU should be taken with caution.

### TWAS

We used TWAS to evaluate predicted changes in differential gene expression in the brain. We identified 23 independent associated genes in nine different brain regions (Supplementary Table S12). The most significant gene was *NPIPB7* (*p* = 1.32 × 10^−10^) with positive enrichment in the cerebellum. Five genes, including *TUFM* (hippocampus), *NPC* (basal ganglia), *SH2B1* (cerebellum), *APOBR* (cerebellar hemisphere) and *CADM2* (cerebellar hemisphere) had a significant effect in the TWAS and also in the GWAS and/or gene-based analysis.

### Fine-mapping of genomic risk regions

We found five genes with predicted causal effects on CanLU in a brain region-dependent manner. We found that higher expression of *NPIPB7* and *PCDH7* in the cerebellar hemisphere and of *NPIPB7* and *LINC02506* in the hypothalamus is potentially related to increased risk for CanLU, as is lower expression of *CADM2-AS1* in the anterior cingulate cortex (ACC; Supplementary Table S13).

### PheWAS

**Fig. 3:**
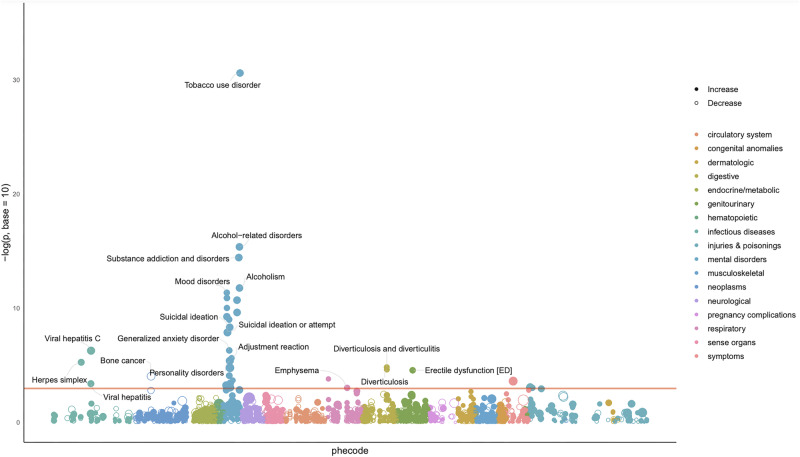
CanLU PRS PheWAS in MGBB. The phecode matrix includes 1711 phecodes. The red line marks the FDR-corrected significance threshold.

Twenty-four traits had a significant association with CanLU (at a threshold of *p* = 2.92 × 10^−5^), all in a positive direction. Of those, 19 are related to mental disorders, while the other traits are infectious, digestive and genitourinary-related traits. The strongest associations were with tobacco use disorder (*p* = 2.51 × 10^−31^), alcohol-related disorders (*p* = 4.56 × 10^−16^) and substance addiction and disorders (*p* = 3.67 × 10^−15^) (Fig. 3, Supplementary Table S14).

## Discussion

In this study, we aimed to evaluate the genetic architecture of CanLU, initially among subjects from the AoU biobank. We previously showed that CanLU differs genetically from CanUD [17]. Probably due to inadequate power, we found no statistically significant effect of any locus in the available non-EUR ancestries (AFR, AMR, SAS, EAS). There were also no significant loci in the individual-sex analysis of EUR in AoU; however, strong genetic correlation between males and females suggest a similar genetic mechanism underlies CanLU in both sexes. In EUR subjects we found a significant effect of CADM2*rs1821351.

We then conducted a meta-analysis of the AoU EUR subjects with previously published EUR summary statistics from ICC and UKBB [14], yielding a total of 271,134 participants (106,680 cases). In addition, we meta-analyzed the entire cross ancestry sample (including the meta-analyzed EUR, and AFR, AMR, EAS and SAS subjects from AoU). Eleven and 15 independent lead SNPs were associated with CanLU in the EUR and cross-ancestry meta-analyses, respectively, with all but three of these loci – CADM2*rs7609594, NCAM1*rs9919557 and rs2106480 – novel for this trait. None of these 15 loci were significant for CanUD [17].

The strongest hits were CADM2*rs7609594 in EUR (*p* = 7.4 ×10^−20^) and CADM2*rs1821351 cross-ancestry (*p* = 7.34 ×10^−19^), replicating previous findings of this gene’s association with CanLU [14, 15], with greater significance than in previous studies, confirming an important role for *CADM2* in CanLU. *CADM2* has also been associated with other substance use traits (alcohol, opioid, cocaine) [18, 19, 55] as well as sexual behavior, risk taking [18, 20] and impulsivity [21], indicating a role in behavioral phenomena that, like cannabis use, are conceptually associated with addiction, risk-taking and openness to experience. TWAS analysis revealed that CanLU is associated with upregulation of *CADM2* in the cerebellar hemisphere, and fine-mapping revealed downregulation of *CADM2-AS1* in the ACC. ACC and cerebellar hemisphere are both involved in substance use [56, 57]. The various roles played by *CADM2* – from insulin sensitivity and energy expenditure [58] to executive functioning and memory [59] – suggest that its expression in different brain regions may affect substance use in multiple ways. As an antisense RNA molecule, CADM2-AS1 may play a role in CADM2 regulation, but we did not find studies that confirm such an association. A recent gastric cancer study found that *CADM2-AS1* is aberrantly expressed in cancer lymph node metastatic tissues, where it promotes metastasis through interaction with miR-5047 and activating NOTCH4 translation, indicating a biological process not directly linked to CADM2 per se [60]. We found a significant genetic correlations between CanLU and behaviors such as sexual behaviors, alcohol-related- and smoking-related-traits, CanUD, OUD, self-harm, risk-taking and openness to experience. Among these traits, six were locally genetically correlated with CanLU in a region on chromosome 3 that includes *CADM2*, further supporting the involvement of this gene in risk-taking. These results are in line with previous findings of local genetic correlation between CanLU and CanUD, as well as between CanUD and smoking initiation and CanUD and alcohol consumption in a region that includes *CADM2* [22].

Nine of these traits had a bidirectional causal relationship with CanLU, providing further evidence for the strong genetic underpinning of different substance use and risk-taking behaviors. While for some of these relationships the bidirectional effects may seem counterintuitive – e.g., CanUD cannot precede CanLU – causality as calculated using MR is not a phenotypic measure, but rather an estimate derived from genetic instruments, i.e. it tests whether having the genetic variants which are associated with the liability to develop CanUD – i.e., variants that predict increased risk for CanUD on a population level – increases the likelihood of CanLU, suggesting a potential causal relationship at the level of genetic liability. We should, however, consider some of these results (namely the causal associations with self-harm, lifetime smoking, lifetime number of sex partners, PAU and weekly alcohol intake) with caution due to potential horizontal pleiotropy.

These associations gained further support in the PheWAS analysis, which revealed a significant association between CanLU and 24 different traits – 19 of which are associated with mental disorders, the three strongest associations being with substance use traits. One of the more interesting significant traits from the PheWAS was erectile dysfunction (ED), in line with studies suggesting that ED is twice as prevalent among cannabis users as in the general population [61].

We observed a significant effect of *NCAM1* on CanLU in EUR and cross-ancestry, consistent with an effect found for this gene in a smaller dataset of CanLU in EUR subjects [14]*. NCAM1* encodes neural cell adhesion molecule 1, and was associated with nicotine dependence [62], alcohol dependence [63] and in multi-trait analyses [64]. Its map position is close to that of *DRD2*. It also contributed to the genetic correlation between cannabis and cigarette smoking and schizophrenia [65], though in our study we did not find a local genetic correlation between CanLU and schizophrenia near *NCAM1*. We did, however, find a local genetic correlation between CanLU and lifetime smoking in this region, again supporting the involvement of *NCAM1* in both traits.

In EUR, we identified a significant effect of *PCDH7*, which may play a role in tumor development [66, 67], on CanLU. Two GWS SNPs were identified on chromosome 2, located near *LRRTM2* and *NRXN1*. Both genes encode proteins that regulate excitatory synapse formation [68], and *LRRTM2* was also associated with CanLU in a MAGMA analysis [14]. Animal studies demonstrate that *LRRTM2* plays an important role in hippocampal connectivity [69, 70], and genetic studies show that *NRXN1* is involved in a variety of neuropsychiatric traits, including schizophrenia [71, 72], nicotine dependence [73] and risk of suicide [74].

We found a significant effect for *NPC*, encoding the Niemann–Pick type C1 protein, an intracellular cholesterol transporter associated with obesity, BMI and diabetes [75, 76]. Nevertheless, we did not find a genetic correlation between CanLU and BMI, nor with 10 out of 11 cardiovascular traits measured – other than a negative genetic correlation with systolic blood pressure (BP). The local genetic correlations between some of these traits and CanLU were not close to the region on chromosome 18 where *NPC1* maps. However, Niemann-Pick disease type C, a disease caused by mutations in the *NPC1* and *NPC2* genes [77, 78], is accompanied by psychiatric symptoms – mainly psychotic, mood-related, and impulse control-related. Also on chromosome 18, we found a significant effect of *DCC*, which has been associated with risk of depression [79], schizophrenia [80] and smoking initiation [23]. *AS3MT*, which has also been associated with schizophrenia [81, 82], was significant for CanLU both in EUR and cross-ancestry.

*MSANTD1* had a significant effect in the cross-ancestry GWAS, in accordance with a previous MAGMA analysis that associated it with CanLU [14]. This gene had an effect on alcohol consumption frequency [23] and educational attainment [83] too. The latter trait was also associated with *SND1* and *AKT3* [83], two genes that were significant in our cross-ancestry analysis, and were also significant for risk taking behavior [20]. *AKT3* was also associated with smoking initiation [23]. In the TWAS and fine-mapping analyses, the most prominent findings point to a causal effect of *NPIPB7*, suggesting that higher expression of this gene in the cerebellum may lead to CanLU. This is a gene that was previously associated with intelligence [84].

Of 43 traits considered, 22 traits were genetically correlated with CanLU in EUR, including 11 related to risk taking, sexual behavior, openness to experience, and substance use, consistent with previously published findings [14, 22, 85, 86]. Of these, nine pairs were positively correlated – only age of first sexual relations and age of first smoking a cigarette were negatively correlated – suggesting that younger age of experiencing these behaviors was associated with CanLU, consistent with the same trend of higher openness to experience among cannabis users.

There are conceptual similarities between CanLU and lifetime tobacco smoking (i.e., when the means of intake for cannabis is smoking it, both are lifetime smoking traits, differing by substance; many cannabis lifetime users are also lifetime cigarette smokers) [87], which are embodied in the strong genetic correlation we found between CanLU and lifetime smoking, and the strong association found in the pheWAS between CanLU and tobacco use disorder (the strongest effect in this analysis). Therefore, we examined the genetic correlations between lifetime smoking and all the traits that were analyzed for genetic correlation with CanLU. For traits that CanLU was genetically correlated with, most of the relationships were similar. Nevertheless, we found a positive correlation between CanLU and educational attainment, which contrasts with the negative association between educational attainment and lifetime smoking. Previous studies have also shown a positive relationship between educational attainment and CanLU [14, 17] and a negative association between education and smoking [88, 89]. These differences might be attributed to different social attitudes towards cigarettes and cannabis reflected in how individuals are attracted to psychoactive substances that they use: cigarette smoking has been considered unhealthy and dangerous for the last six decades, while cannabis is perceived by the public as generally safe [90] and is now even being used for medical purposes, albeit with limited empirical support. An educated person might know that cigarettes are unhealthy but be less informed regarding the harmful effects of cannabis, a possibility supported by a causal effect we found for educational attainment on CanLU, but not the other way around. Our findings are based mostly on adult participants (>18 in AoU, >16 in UKBB and ICC); in adolescents, the relationship between cannabis use and educational attainment is negative [91, 92]. Another difference is that unlike CanLU, lifetime smoking was associated with lung cancer and with cardiovascular disorders, in line with well-established knowledge regarding the hazards of cigarette smoking [93], and suggesting possible differences between the harmful effects of smoking tobacco vs cannabis.

However, some of the differences that were seen between lifetime cigarette smoking and CanLU regarding their genetic correlations with other traits, were also seen when we compared CanLU with CanUD. Like lifetime smoking, CanUD was genetically correlated with cardiovascular diseases and lung cancer, and had a negative r_g_ with academic degree. The latter finding is consistent with previous findings [17]. Unlike CanLU, CanUD was also associated with sleep-related traits, in line with known association between chronic cannabis use and sleeping disturbances [94]. Although CanLU had a moderate genetic correlation with CanUD (r_g_ = 0.58), none of the genes identified in our GWAS nor in the MAGMA analysis had a significant effect in the largest GWAS of CanUD published to date [17], providing additional evidence of the genetic differences between CanLU and CanUD, and supporting the need to study these traits separately.

We found a positive genetic correlation between CanLU and schizophrenia, with MR indicating that schizophrenia is causal for CanLU but not vice versa. This differs from the bidirectional causal relationship found between schizophrenia and CanUD [17], suggesting that genetic liability to use cannabis in the absence of liability to dependence may not be considered a risk factor for schizophrenia, but that genetic liability to schizophrenia might lead to cannabis use initiation. Four cardiovascular traits had local genetic correlations with CanLU (between 1-3 regions), but only systolic BP had a significant – negative – effect when measured using LDSC. Literature regarding the interaction between cannabis use and BP is inconclusive, as some studies show that cannabis use reduces systolic and diastolic BP [95] and others suggest an elevation in systolic, but not diastolic, BP due to cannabis use [96, 97]. Therefore, further studies of the relationship between cannabis use and BP are needed.

We found a strong genetic correlation between male and female CanLU, suggesting that the genetic factors that influence CanLU are similar across sexes. These results are in line with high male-female r_g_ values found in studies of other substance use traits such as alcohol consumption (r_g_ = 0.81–0.9) [98, 99] and alcohol use disorder (AUD) (r_g_ = 0.83) [99].

This study is the largest analysis to date of the genetic basis of CanLU. We point to 11 independent GWS loci associated with CanLU in EUR, eight of which in protein-coding regions. Cross-ancestry, we found 15 lead SNPs, ten in protein-coding regions. In total, 13 protein-coding genes were associated with CanLU. Out of those, six were previously associated with CanLU through GWAS and/or MAGMA analyses: *CADM2*, *NCAM1*, *MSANTD1*, *LRRTM2*, *NPC1* and *AS3MT* [14]. Of the other seven, six were associated with at least one other substance use trait, mostly alcohol- and smoking-related, including *AKT3*, *DCC*, *NRXN1*, *PCDH7*, *SND1* and *PTPN11* [23, 100]. To our knowledge, *RAI1* is the only gene that was significant in our study of CanLU and was never previously associated with any substance use trait. *CADM2* had the strongest effect in our study, consistent with previous findings [14, 15]. We also show a broad genetic correlation between CanLU and other traits related to openness to experience and risk taking, including substance use behaviors, sexual behaviors and personality traits. Most of these traits had a bidirectional causal interaction with CanLU, and six were locally genetically correlated with CanLU in a region that maps to *CADM2*. Although enhanced power led to more GWS loci in our study than in any other previous GWAS of CanLU, there are several important similarities with all these studies. Besides the replication of the GWS effects of *CADM2* and *NCAM1*, several other genes that had a significant effect in a previously published gene-based analysis of CanLU [14] were also significant in our GWAS (*AS3MT*, *NPC1*) and/or MAGMA (*CLN3*, *APOBR*, *IL27*, *CCDC101*, *ATXN2L*, *TUFM*, *SH2B1*, *ATP2A1*, *C18orf8*, *NPC1*) analyses. The genetic correlations we found between CanLU and sexual behaviors, substance use and other forms of risk taking are similar in magnitude and direction to previous findings [14, 16]. In all, these results emphasize the replications observed in this study, pointing to the consistency observed across differently-ascertaining samples of the genetics of CanLU and to potential generalizability of our results.

This study has limitations. First, we considered CanLU as a unitary trait, with no distinction between subjects who used cannabis only once or a few times in their lives and regular cannabis users, including those with CanUD. Differences in routes of cannabis administration (e.g. smoking, eating), cannabis strains, and active components of the plant (e.g. phytocannabinoids) could not be considered given the nature of the available datasets. Second, the use of cannabis often co-occurs with that of other substances, a characteristic that we did not control for in our study. Therefore, some of the results may be influenced by polysubstance use and not be specific to CanLU. Third, because most subjects in this study were of EUR ancestry, there was not enough power to detect GWS loci in other ancestries when analyzed independently in AoU. Similarly, the MGBB biobank includes too few non-EUR subjects to conduct a meaningful pheWAS, as we did in EUR. As more data for non-EUR ancestries become available, it will be possible to detect genomic loci associated with CanLU in these populations, and conduct additional meaningful post-GWAS analyses as well. Fourth, this study was limited to common variants, and future studies which will include rare variants may lead to a deeper understanding of the genetics of CanLU. In addition, there were case-control ratio differences among the different cohorts that were analyzed in this study (58% cases in AoU, 43% in ICC, 22% in UKBB) and likely differences in the characteristics of the study participants that might have been consequential. These could reflect differences in the social acceptance of cannabis between the UK and USA, or to the years the data were collected (before/after cannabis medicalization and/or legalization in different jurisdictions), and probably both. With the passage of time, the prevalence of cannabis use is increasing [1], the risk-taking factor of CanLU may be reduced (which may also have implications on future GWAS and future genetic correlation analyses), and the consequences (e.g., increased incidence of CanUD, consistent with the high genetic correlation between CanLU and CanUD) can be harmful. Therefore, ongoing research that can utilize the growth in biobanks and in cannabis use prevalence, is needed to characterize the genetic architecture of cannabis lifetime use and its consequences.

## Supplementary information

Supplementary figures

Supplementary Tables

## Data Availability

Summary statistics will be made available for download on the Gelernter Lab website.
