# Supplementary material for: The genetics of cannabis lifetime use
Source: Neuropsychopharmacology. 2025 Oct 3;51(3):554–64. doi: 10.1038/s41386-025-02255-4 (PMC12824155; doi:10.1038/s41386-025-02255-4)
Supplement: Supplementary file 1 — Supplementary figures [file 41386_2025_2255_MOESM1_ESM.docx]

**
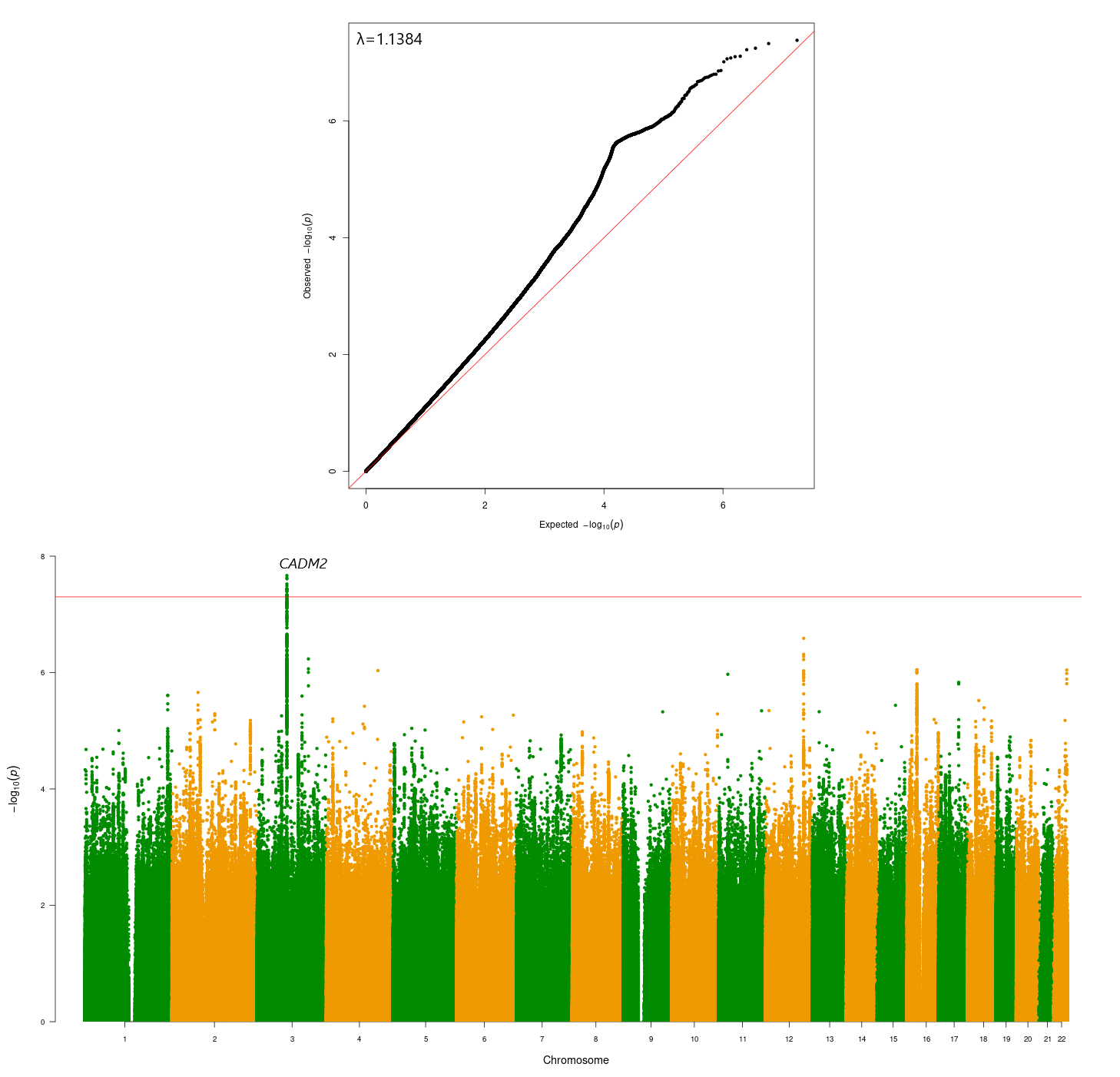
**

**Figure S1:** EUR GWAS (All of Us) of CanLU

**
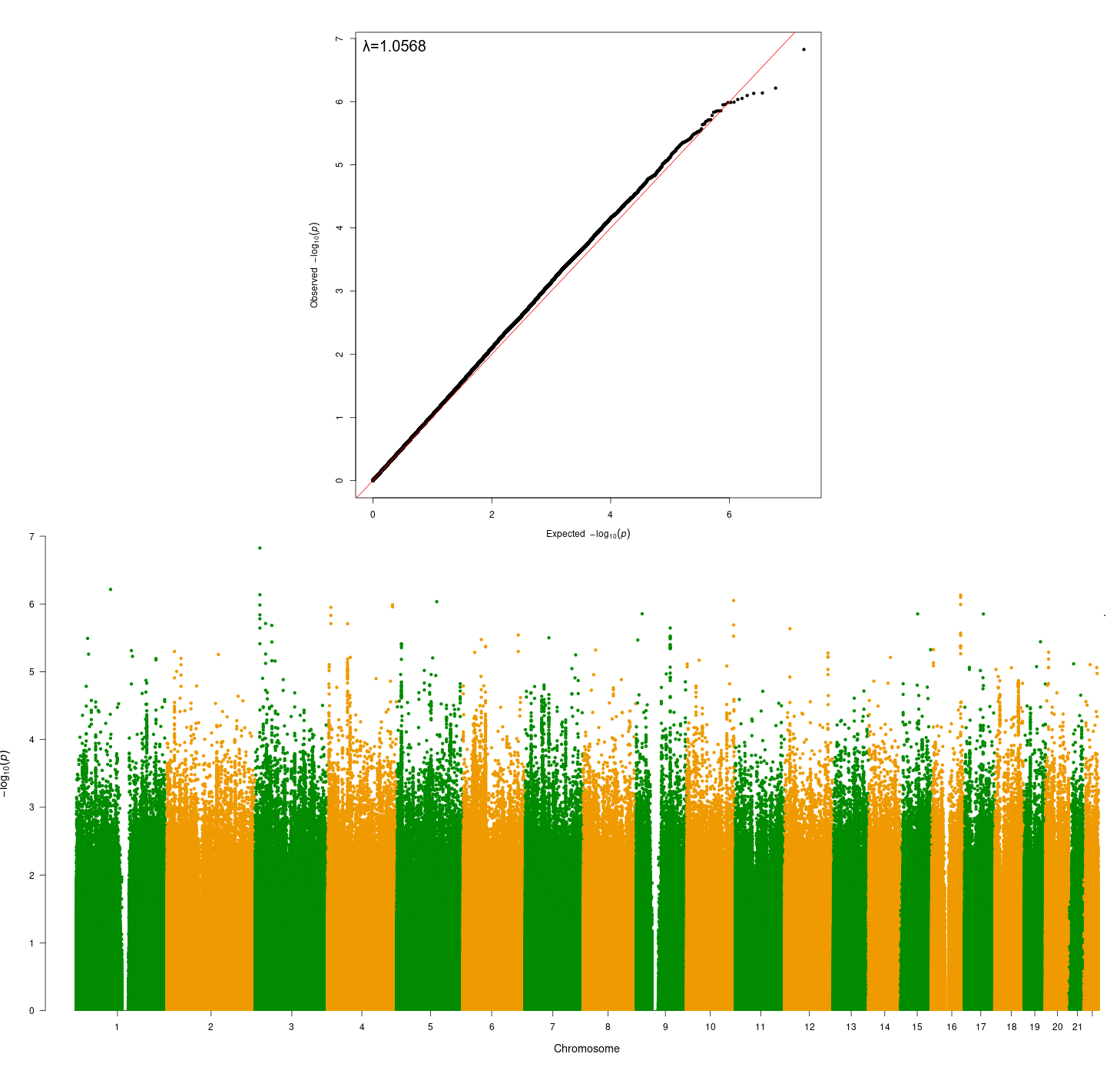
**

**Figure S2:** AFR GWAS (All of Us) of CanLU

**
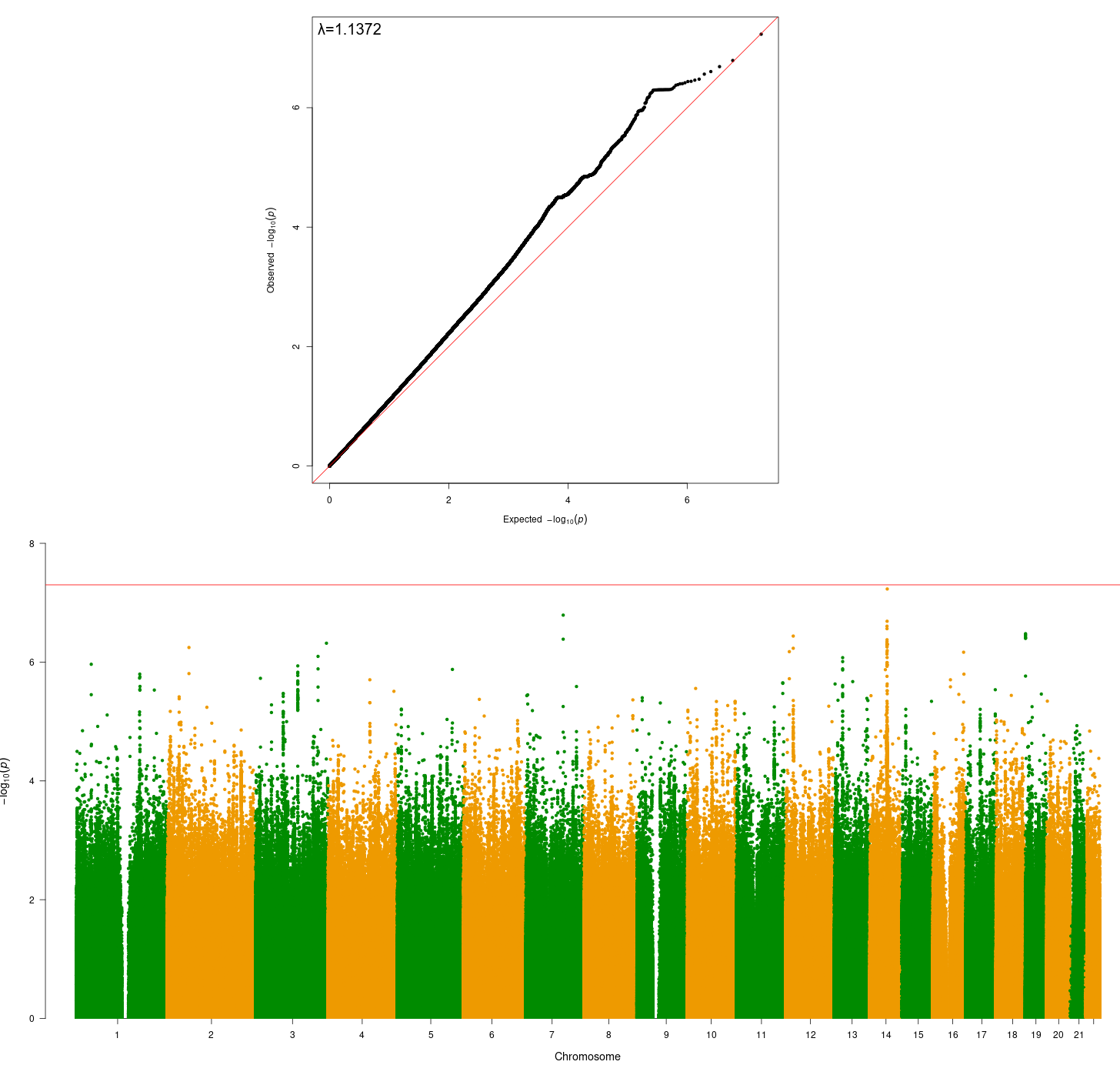
**

**Figure S3:** AMR GWAS (All of Us) of CanLU

**
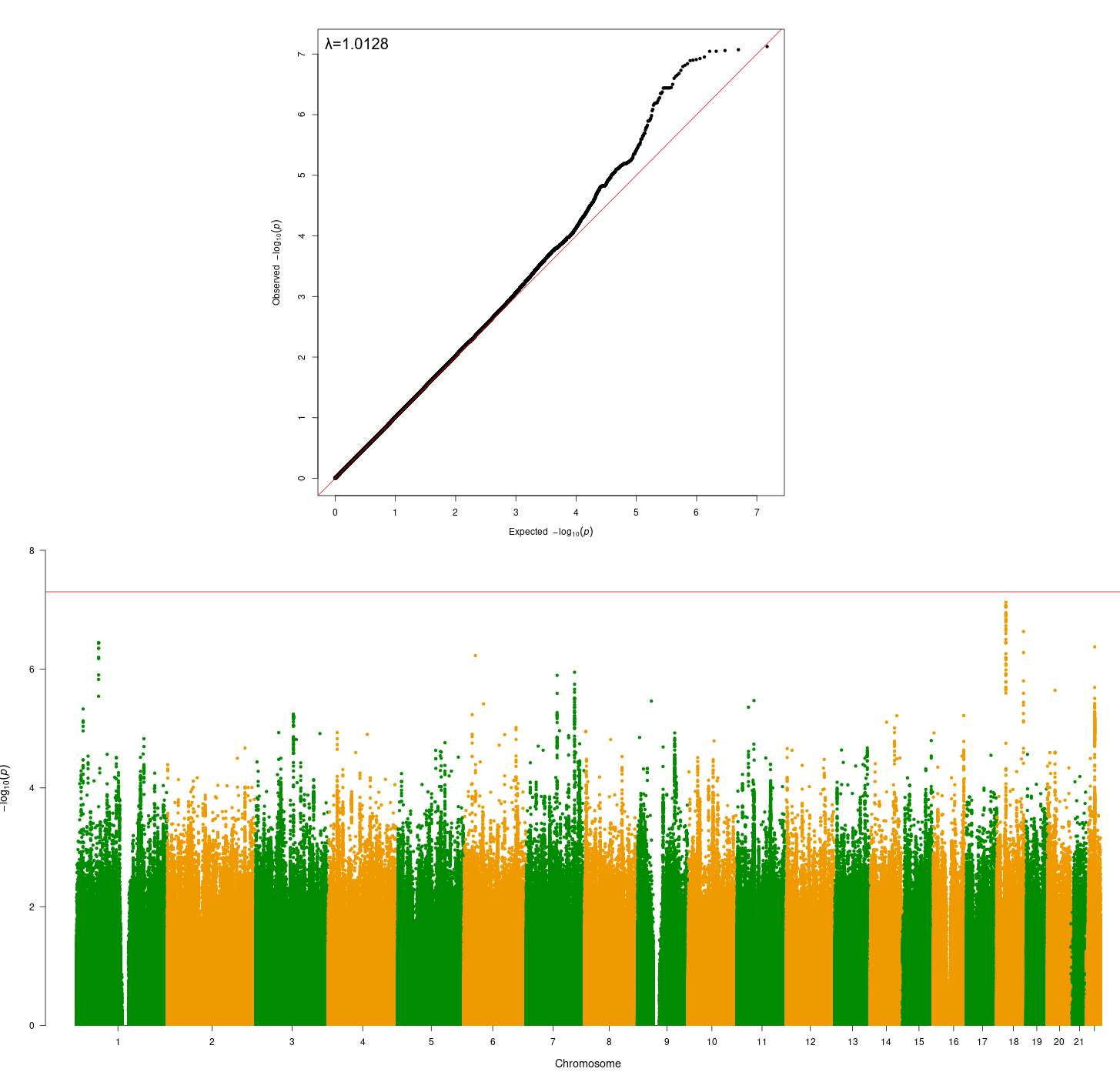
**

**Figure S4:** EAS GWAS (All of Us) of CanLU

**
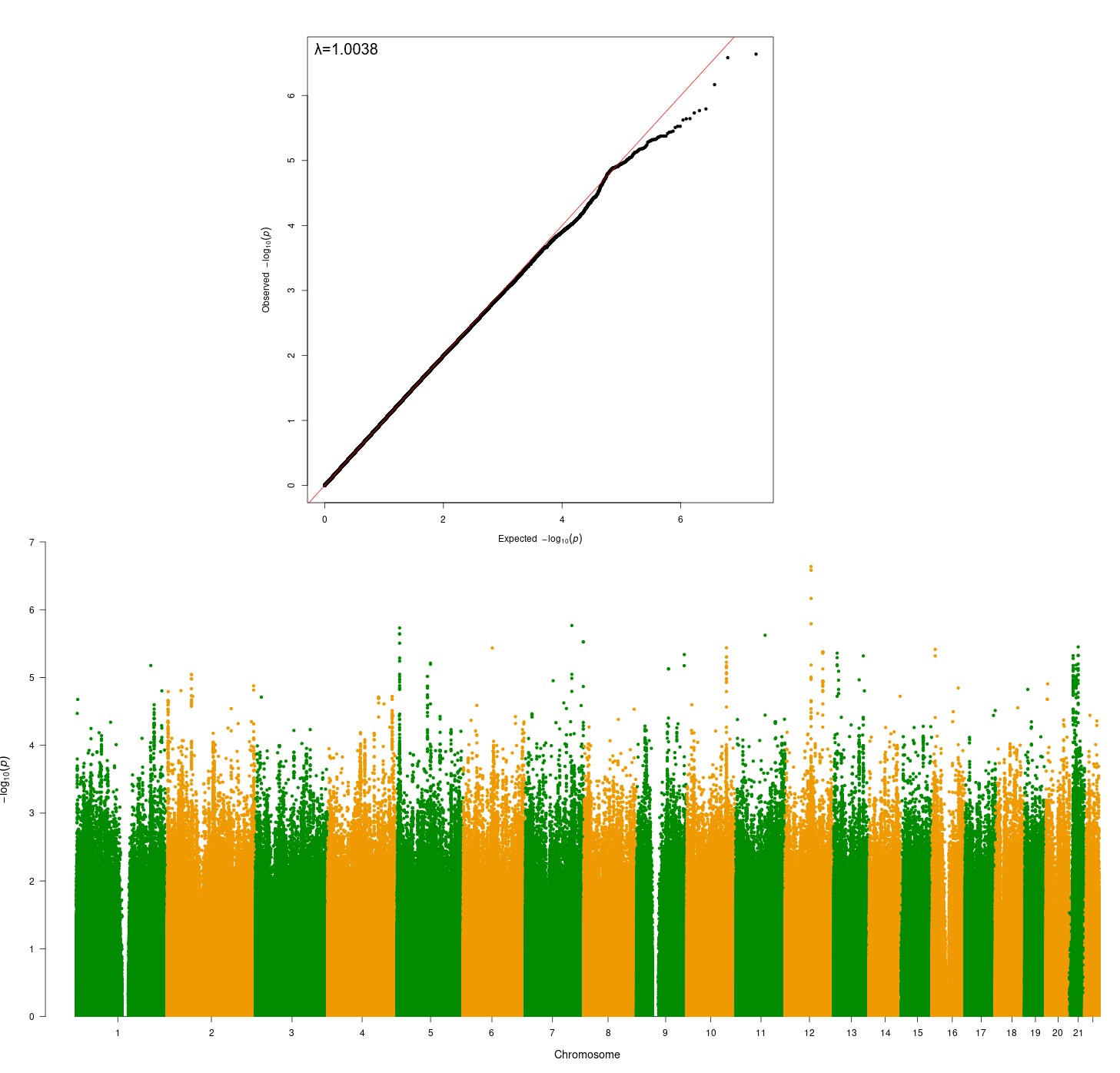
**

**Figure S5:** SAS GWAS (All of Us) of CanLU

**
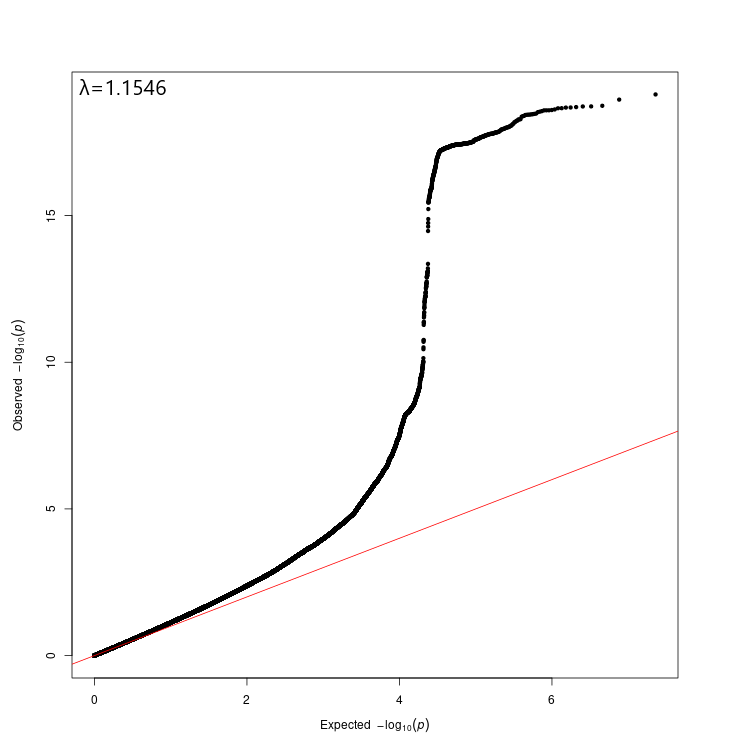
**

**Figure S6:** QQ plot of the EUR GWAS meta-analysis

**
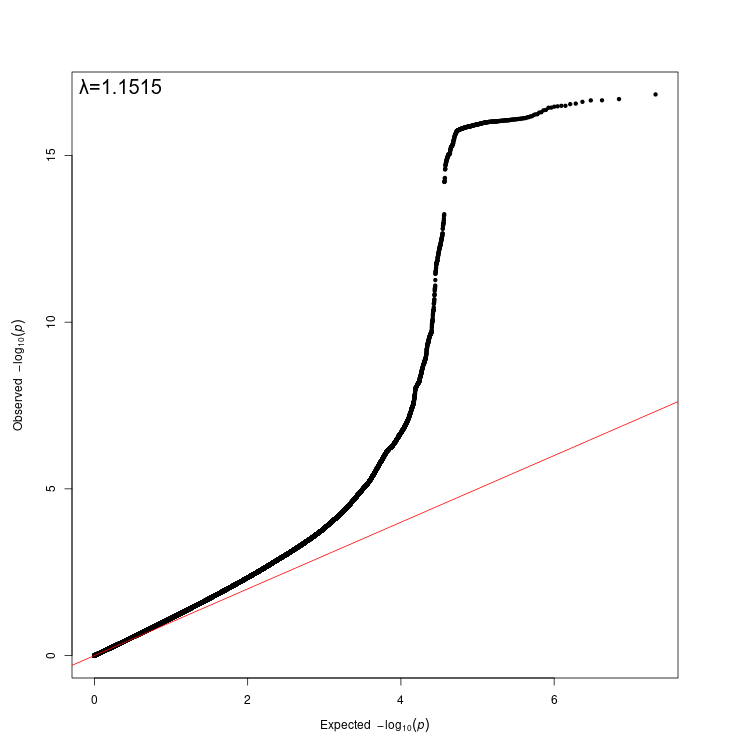
**

**Figure S7:** QQ plot of the cross-ancestry GWAS meta-analysis


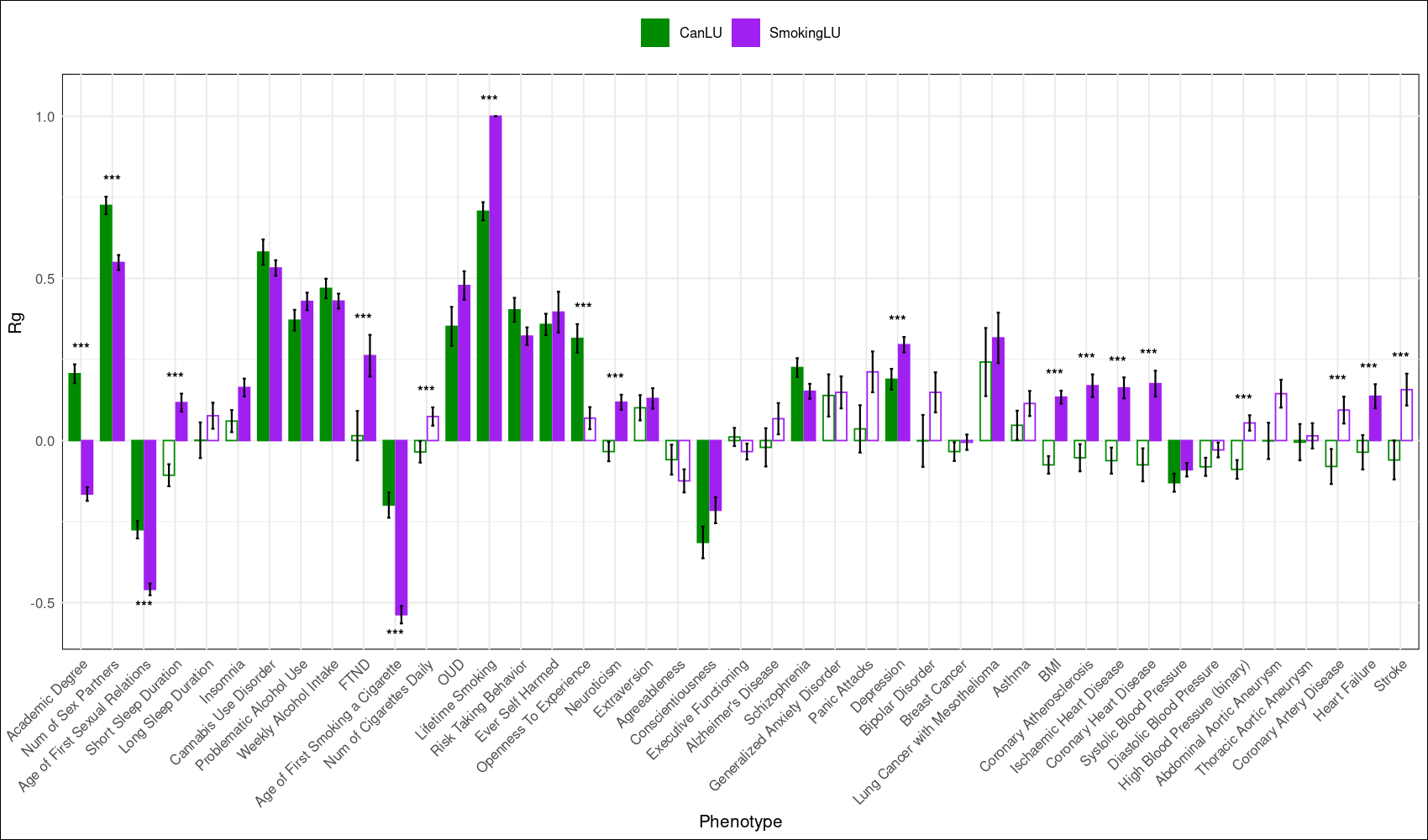


**Figure S8:** Genetic correlations between lifetime smoking (smoking LU) and a list of traits, compared to the genetic correlations between these traits and CanLU (CanLU data is similar to that presented in Figure 2 in the manuscript). Empty bars represent non-significant r_g_ values. For each pair, differences in r_g_ were measured using a Wald test, accounting for the covariance between the estimates (*** p<3.88x10^-4^).

**
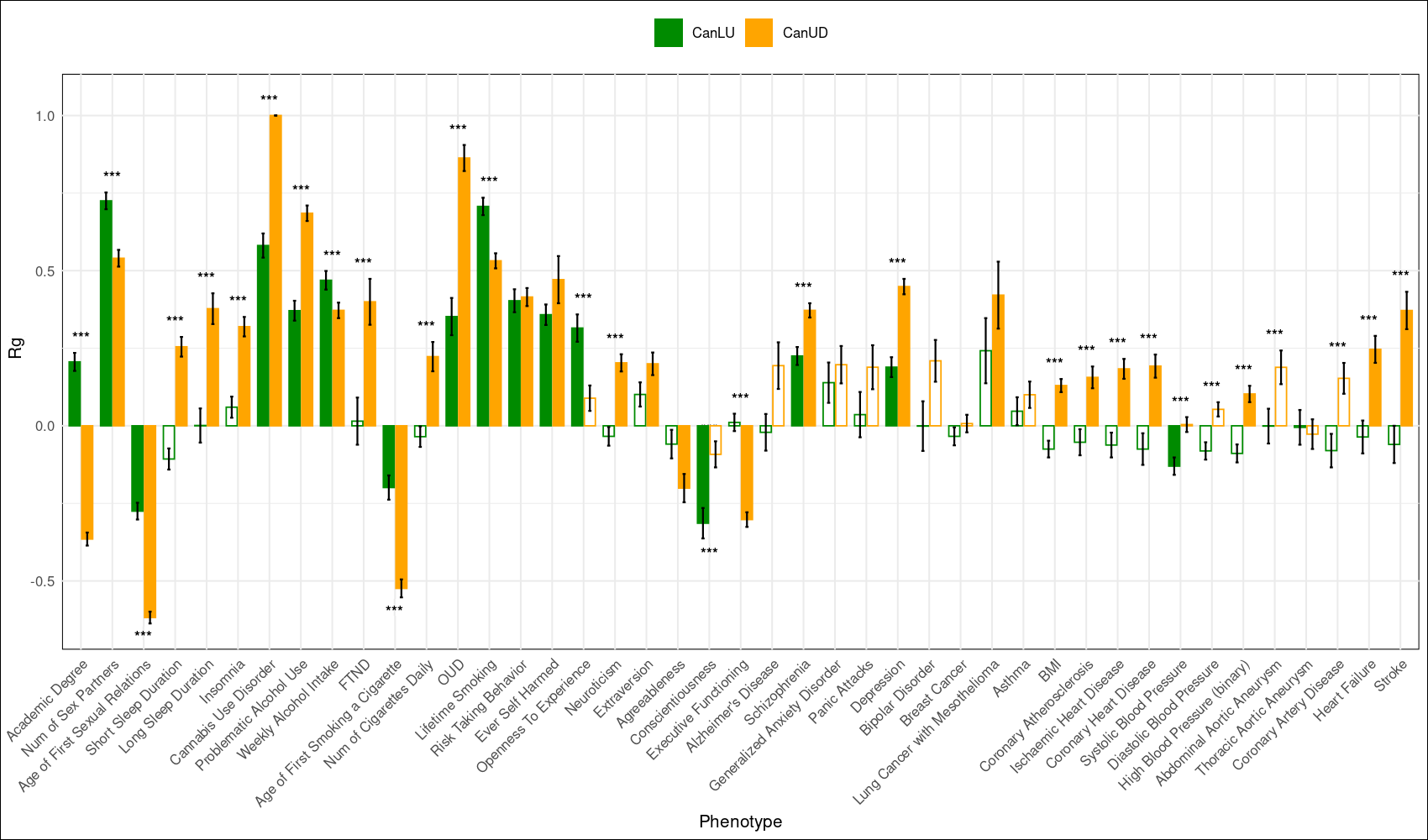
**

**Figure S9:** Genetic correlations between cannabis use disorder (CanUD) and a list of traits, compared to the genetic correlations between these traits and CanLU (CanLU data is similar to that presented in Figure 2 in the manuscript). Empty bars represent non-significant r_g_ values. For each pair, differences in r_g_ were measured using a Wald test, accounting for the covariance between the estimates (*** p<3.88x10^-4^).
